# Bowel Obstruction From Biliary Stent Migration: An Unusual Case of Abdominal Pain

**DOI:** 10.7759/cureus.52257

**Published:** 2024-01-14

**Authors:** Brian G Nudelman, Marianne Cortes, Raphael Khella, Antony George, Camilo Ruiz

**Affiliations:** 1 Internal Medicine, Memorial Healthcare Pembroke, Pembroke Pines, USA; 2 Osteopathic Medicine, Nova Southeastern University Dr. Kiran C. Patel College of Osteopathic Medicine, Davie, USA; 3 Internal Medicine, Florida Atlantic University Charles E. Schmidt College of Medicine, Boca Raton, USA; 4 Internal Medicine, Broward Health Medical Center, Fort Lauderdale, USA

**Keywords:** sbo, ercp, mrcp, endoscopic, common bile duct, cholecystitis, stent, gi, gastroenterology, biliary

## Abstract

Endoscopic biliary stent placement is an important procedure that is commonly done in patients with malignant obstruction of the biliary tree. However, it can also be done to relieve non-maligant obstructions short term until more curative surgical interventions can be performed. There are two main types of stents used for these procedures: self-expanding metal stents (SEMSs) and plastic stents. Each of these stent types has different indications, and determining the correct stent for each individual patient is important. Here, we present a case of a 73-year-old female who presented with abdominal pain due to small bowel obstruction caused by a dislodged biliary duct stent. We hope to promote more focus on selecting the right stent type for each patient and encouraging follow-up visits after placement, especially for those with a history of medical noncompliance.

## Introduction

Endoscopic stenting is a procedure that is often performed to manage cholangitis, symptomatic jaundice with pruritus, delayed surgery, and biliary obstruction [1]. Sometimes, these obstructions can be caused by malignancy. Malignant tumors that may cause an obstruction include, but are not limited to, cholangiocarcinoma, periampullary tumors, and metastasis from other cancers like hepatocellular carcinoma. The obstruction can occur either at the proximal or distal ends of the bile ducts. There are several reasons for a patient to receive a biliary stent. It could be placed for the management of symptoms, such as pruritus, or to treat cholangitis in patients with cancers that cannot be surgically cured, with the stent providing relief from cholestasis. Stenting may also be performed in patients who are awaiting surgical resection of a tumor if they have cholangitis or if they need surgery in the near future. The stents themselves may either be plastic or metallic, with the latter being more common. Plastic stents are mainly utilized as a bridge to surgery, but metal stents are usually used in inoperable cases. Self-expanding metallic stents (SEMSs) come either bare, partially, or fully covered. Using an uncovered SEMS is advantageous in that they can be placed in any location of the biliary tree, and they have been shown to migrate less often in studies. The theory behind the less frequent migration is that eventually the metal stent becomes embedded in the tissue. This in turn is an advantage of covered SEMSs in that they are removed with greater ease. There is an array of metal stents that have different sizes and methods of insertion with diameters ranging from 5 to 10 mm [2]. 

There are several complications that can occur following a biliary stent placement including obstruction of the stent and cholangitis, but biliary stent migration is a rare complication that can occur as well. Wide sphincterotomies and abnormal dilation of the biliary system can precipitate migration of the stent following placement as the stent may have more room to travel [3].

## Case presentation

We present a case of a 73-year old African American female who presented to the emergency department with a chief complaint of crampy abdominal pain, which was increasing in severity over the previous 24 hours. She complained of constipation and increased flatulence but denied nausea, vomiting, diarrhea, and blood per rectum. Of note, the patient was a poor historian with a very low medical literacy and was only able to give minimal details about her medications, past surgical history, and medical history. She did not follow regularly with a primary care physician (PCP), and attempts to gather history from family members were unsuccessful. From the patient's electronic medical chart, we found that she had a past medical history of hypertension, human immunodeficiency virus, and chronic hip pain. She also had a surgical history of a laparoscopic ovarian cystectomy 10 years ago and a total left hip arthroplasty nine years ago. The patient denied any history of cancer or abdominal surgery and denied recollection of any other procedures, including endoscopic retrograde cholangiopancreatography (ERCP).

On the initial exam, the patient was malnourished-appearing and in mild distress due to abdominal tenderness. She was afebrile with a heart rate of 80 beats per minute, blood pressure of 150/90, respiratory rate of 18 breaths per minute, and oxygen saturation of 99% on room air. Her lungs were clear, and no cardiac murmurs were present. Bowel sounds were normal in all four quadrants. Upon palpation, her abdomen was vaguely tense, and she showed slight guarding but denied rebound tenderness. Her abdominal pain was localized to the epigastric region and then relocated to the umbilical region over the proceeding hours. Labs on admission showed mild elevation in both liver function tests (LFTs) and white blood cell (WBC) count with all other labs, such as WNL. The patient underwent kidney, ureter, and bladder (KUB) X-ray, which revealed moderate dilatation of the small bowel loops in the left side of the abdomen with marked stool in the right colon. Follow-up CT abdomen/pelvis with IV contrast revealed nondilated loops of fluid-filled small bowel and pneumobilia with dilatation of the intrahepatic bile ducts.

**Figure 1:**
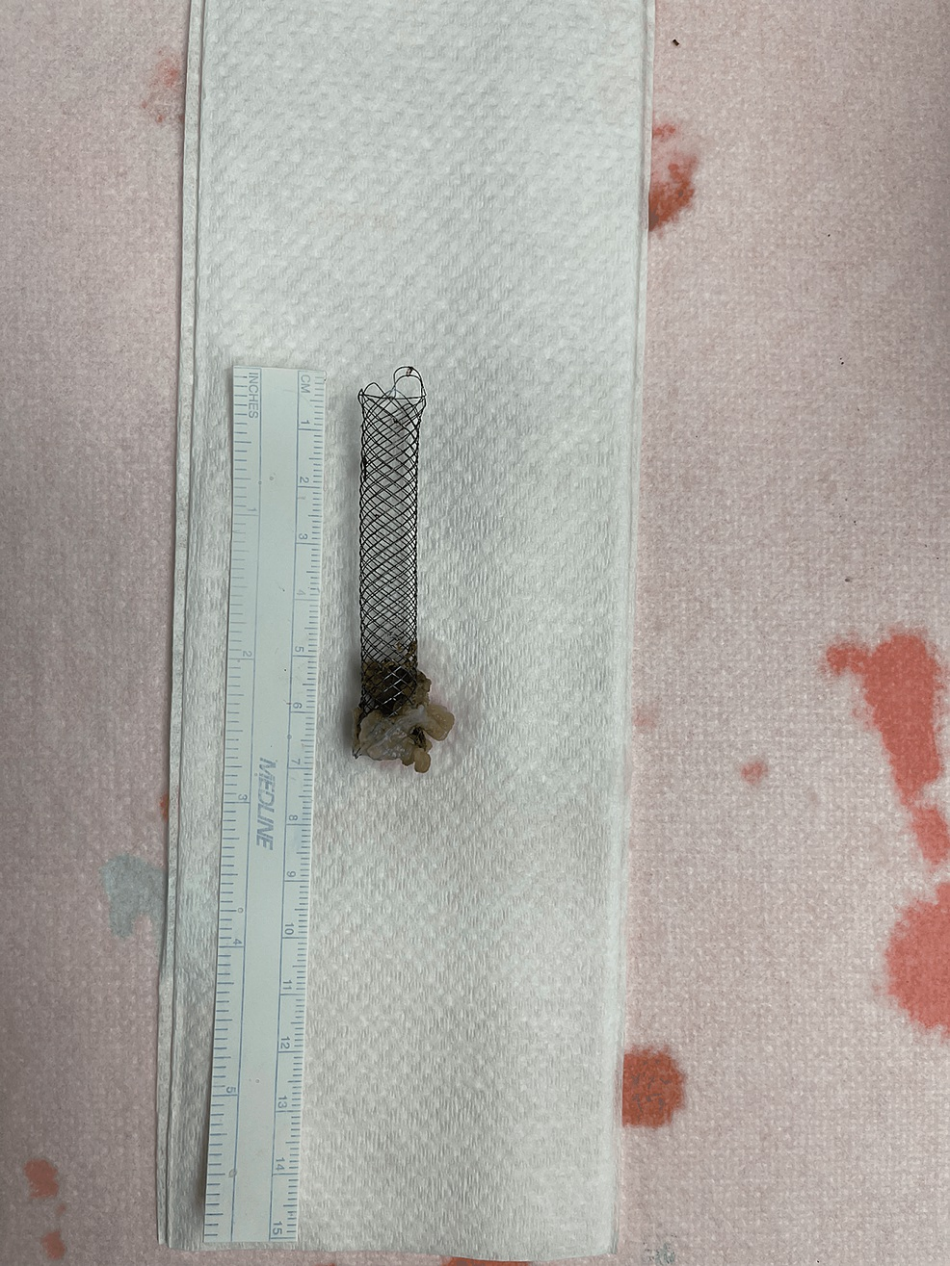
A stent measuring 1x6 cm removed from the ileum

The patient was admitted and made NPO ("nothing by mouth"), an nasogastric (NG) tube was placed to low intermittent suction, and general surgery was consulted. Upon further review of the imaging, it appeared that there was a stent-like foreign body that measured 1x6 cm, which had migrated into the small bowel in the area of the right lower quadrant. The patient was taken to the OR emergently, where she underwent laparoscopic lysis of adhesions with conversion to open for small-bowel resection with primary anastomosis and removal of the foreign body. The foreign body was found embedded in the ileum wall at an area with significant narrowing secondary to adhesions. The rest of the small bowel and large bowel looked well with no strictures or perforations. The foreign body in question was sent to pathology, and ultimately was determined to be a stent, which had embedded in the wall of the small bowel (Figure 1). A peripherally inserted central catheter (PICC) line was inserted to provide total parenteral nutrition (TPN) until a follow-up small bowel series could be performed to evaluate for post-operative anastomotic leak or possible missed bowel perforation.

Small bowel series was unremarkable, and the patient's diet was slowly advanced. She was ultimately placed on a gastrointestinal (GI) soft diet, which she continued with at the time of discharge. Discharge planning was discussed with the patient's children, who determined that the best course of action for their mother was to have her discharged to a skilled nursing facility. She was accepted to a local inpatient rehab.

Regarding the patient's findings of pneumobilia, follow-up limited abdominal ultrasound revealed pneumobilia with gallbladder sludge. GI was consulted, and the patient subsequently underwent MRCP, which revealed distended gallbladder with bile, minimal sludge without gallstones, and pneumobilia involving the intra- and extrahepatic biliary system and gallbladder. Repeat ultrasound revealed stable findings, and the patient was cleared for discharge. She was discharged to a skilled nursing facility (SNF) with instructions to follow up with General Surgery and Palliative Care. All questions and concerns were addressed prior to discharge.

## Discussion

This presentation demonstrates an interesting case of biliary stent migration in an elderly patient. Although the most common complications from biliary stent placement are usually stent obstruction and cholangitis, stent migration can happen on rare occasions. Migration of a stent outside of the common biliary duct can happen due to biliary dilatation, prolonged duration of stent insertion due to neglect, and wide sphincterotomy at the sphincter of Oddi. A retrospective study from 2021 aiming to determine the frequency and risk factors for biliary stent migration showed that out of 876 patients, 8.4% had their biliary stent migrate. Interestingly, it was found that leaving a biliary stent in for more than one month increased the risk that it would dislodge and migrate. In our patient’s situation, a combination of patient negligence and possible lack of patient education by the ERCP physician led to neglect of her stent and eventual migration to the ileum where it caused her pain. While the most common adverse effects of stent migration seen in the study were cholangitis and stent obstruction, there were two cases of duodenal bleeding and two cases of duodenal wall rupture seen as well. This is important to note as a migrated stent could become a real medical emergency. In addition, in two patients, the stent migrated to the colon, and in one patient, the stent migrated to the area of the ileocecal valve causing incomplete obstruction [1,3]. This presentation is very similar to the one we observed with our patient.

In a literature review from 2014, researchers compiled data from multiple case reports in which patients had ERCP with stent placement but then were lost to follow-up. The normal length of time for stent placement and removal or replacement is about three to six months, but sometimes, stents are unintentionally left in longer [4]. Through their research, they found that stents placed for longer than three years and stents that dislodged into the common bile duct were two of the biggest risk factors for "forgotten" stents. They proposed that all stents have a registry system in which they could be appropriately tracked to avoid stent neglect [5]. This would have been very beneficial to a patient like ours that did not maintain follow-up with her GI after her stent placement. 

Most dislodged biliary stents pass through the entire small bowel and colon and are excreted with feces. However, bowel perforation, obstruction, and chronic diarrhea can happen if the patient in question has risk factors that would impede a successful passage of the stent through the GI tract. These risk factors include adhesions from previous abdominal surgery, hernias, and diverticular disease [3]. Our patient's significant surgical history of a laparoscopic ovarian cystectomy put her at risk of abdominal adhesions. The presence of adhesions was confirmed during her laparoscopic surgery, which required conversion to an open small bowel resection with anastomosis. 

Differences in stent type and length have proven to have an effect on the likelihood of stent migration. A 2020 case report with a literature review showed that all reported cases of stent migration in the literature were straight-type stents and not the double-pigtail stents that are less prone to distal migration because they anchor into the biliary tract [6]. Moreover, the use of uncovered SEMSs vs. plastic stents can decrease the risk of migration because the uncovered SEMSs imbed themselves into the biliary tract. This causes them to be significantly harder to remove than plastic stents [3]. That said, plastic stents still have indications in which they are the more favorable option despite increased chance of migration. This is especially true in regards to short-term stent placement in anticipation of another operation. The following table compares the differences between the various types of biliary stents (Table 1) [7].

**Table 1:** Biliary stent comparison SEMS: self-expanding metal stent Credit: Lam R and Muniraj T [7]

Type of stent	Cost	Risk of migration	Removability
Plastic stent	Low	Intermediate/high	Easy
Uncovered SEMS	High	Very low	Difficult
Partially covered SEMS	High	Low	Difficult
Fully covered SEMS	High	High	Difficult

While many things can be done to try and increase patient compliance, none of these methods are guaranteed to be effective. A thorough risk-benefit analysis should be done for each patient with biliary obstruction to determine which stent will be best suited to their situation. In the case of our non-compliant patient, an uncovered SEMS was the best option to prevent dislocation. Based on the specimen that was recovered from her small bowel resection, she did in fact receive a bare SEMS, which is the correct option for her case. We believe that her SEMS may have dislodged because of damage to the sphincter of Oddi during her previous stent placement. Damage to this muscle could have allowed the stent to pass distally into the small bowel. This is supported by the findings of pneumobilia, which could be due to gas entering the biliary tract through the incompetent sphincter. In addition, our patient’s history of laparoscopic surgery left her with adhesions that further complicated her case by not allowing a proper exit of this stent through her GI tract.

## Conclusions

Our case highlights a complication of a dislodged biliary duct stent that had been left in a patient for a prolonged period of time and eventually led to bowel obstruction. We aim to encourage better post-operative education and follow-up practices in patients who undergo ERCP with stenting. Moreover, for those with a previous history of ERCP presenting with small bowel obstruction, stent migration should be included in the differential diagnosis. Furthermore, we conclude that the best stent for those with poor compliance and medical follow-up would be a bare SEMS as it has better adherence to the tissues and lower chance of dislocation.

## References

[1] Dumonceau JM, Tringali A, Papanikolaou IS (2018). Endoscopic biliary stenting: indications, choice of stents, and results: European Society of Gastrointestinal Endoscopy (ESGE) Clinical Guideline - Updated October 2017. Endoscopy.

[2] Meseeha M, Attia M (2023). Biliary stenting. StatPearls [Internet].

[3] Emara MH, Ahmed MH, Mohammed AS, Radwan MI, Mahros AM (2021). Biliary stent migration: why, how, and what?. Eur J Gastroenterol Hepatol.

[4] Namdar T, Raffel AM, Topp SA (2007). Complications and treatment of migrated biliary endoprostheses: a review of the literature. World J Gastroenterol.

[5] Odabasi M, Arslan C, Akbulut S (2014). Long-term effects of forgotten biliary stents: a case series and literature review. Int J Clin Exp Med.

[6] Wang X, Qu J, Li K (2020). Duodenal perforations secondary to a migrated biliary plastic stent successfully treated by endoscope: case-report and review of the literature. BMC Gastroenterol.

[7] Lam R, Muniraj T (2021). Fully covered metal biliary stents: a review of the literature. World J Gastroenterol.

